# Fever and electrocoagulation syndrome after colorectal endoscopic submucosal dissection for patients with immunosuppressants and steroids

**DOI:** 10.1002/deo2.83

**Published:** 2021-12-09

**Authors:** Shumpei Yamamoto, Hideaki Kinugasa, Yasushi Yamasaki, Mami Hirai, Soichiro Ako, Kensuke Takei, Shoko Igawa, Eriko Yasutomi, Shohei Oka, Masayasu Ohmori, Toshihiro Inokuchi, Keita Harada, Sakiko Hiraoka, Kazuhiro Nouso, Takehiro Tanaka, Hiroyuki Okada

**Affiliations:** ^1^ Department of Gastroenterology and Hepatology Okayama University Graduate School of Medicine, Dentistry, and Pharmaceutical Sciences, Okayama University Okayama Japan; ^2^ Department of Pathology Okayama University Graduate School of Medicine, Dentistry, and Pharmaceutical Sciences, Okayama University Okayama Japan

**Keywords:** colorectal ESD, PECS, electrocoagulation syndrome, immunosuppressants and steroids, post‐ESD fever

## Abstract

**Objectives:**

Transient fever and electrocoagulation syndrome after colorectal endoscopic submucosal dissection (ESD) remain a challenge. The aim of this study was to assess the risk factors of post‐ESD fever and post‐ESD coagulation syndrome (PECS), focusing on the involvement of immunosuppressive drugs and steroids (IM).

**Methods:**

This retrospective analysis included 510 patients who underwent colorectal ESD at Okayama University Hospital from 2015 to 2020. The incidence rate, clinical outcome, and factors associated with post‐ESD fever and PECS were investigated.

**Results:**

Post‐ESD fever and PECS occurred in 63 patients (12.4%) and 43 patients (8.4%), respectively. In multivariate analysis, the American Society of Anesthesiologists Physical Status ≥3, the use of immunosuppressants or prednisolone ≥5mg (IM group), and injury to muscle layer/perforation were significantly associated with post‐ESD fever. In PECS, IM group, tumors located on the right side, treatment time ≥60 min, injury to the muscle layer, and multiple lesions were independent risk factors. Both post‐ESD fever and PECS improved conservatively in the IM group, and no serious complication was observed.

**Conclusions:**

The use of IM was a risk factor for both post‐ESD fever and PECS. However, there were no serious complications in colorectal ESD for patients taking IM.

## INTRODUCTION

Colorectal cancer is the second most common cause of cancer‐related mortality worldwide.^1^ Colonoscopy and polypectomy improved the long‐term risk of death from colorectal cancer.^2^ Although most colorectal polyps can be resected by endoscopic mucosal resection (EMR), residual or recurrent neoplasms after piecemeal EMR for large superficial colorectal neoplasms has been a major limitation.^3^, ^4^ Endoscopic submucosal dissection (ESD) has been developed as a new treatment method to address this limitation.

However, although ESD has been suitable for large superficial colorectal neoplasms due to high en bloc resection rates, colorectal ESD has other limitations, such as technical difficulties and a higher incidence of post‐bleeding and perforation complications.^5^, ^6^, ^7^ With the development of new traction methods and submucosal injection agents,^8^, ^9^, ^10^ these weaknesses have been improved, and ESD has become widely used. However, transient fever and electrocoagulation syndrome after colorectal ESD remain a challenge that needs to be addressed.^11^, ^12^, ^13^, ^14^

On the other hand, ESD is less invasive than surgery and is increasingly being chosen as a treatment for patients on immunosuppressants and steroids, because several diseases need immunosuppressants and low‐dose of prednisolone (PSL) (about 5 mg) as maintenance therapy. In clinical practice, we have occasionally encountered postoperative fever in patients with ESD taking immunosuppressive drugs, and we assumed that there might be a relationship between transient fever and immunosuppressants. Immunosuppressants have been reported to have negative effects in several surgical fields, such as the prevalence of infectious complications and influence on wound healing.^15^, ^16^, ^17^, ^18^, ^19^ However, there is no direction that shows the usefulness or risk of ESD for such patients.

In the present study, we assessed the risk factors for post‐ESD fever, post‐ESD coagulation syndrome (PECS), and the postoperative course after colorectal ESD, focusing on the involvement of immunosuppressive drugs and steroids. In addition, we performed a broad‐range 16S rRNA polymerase chain reaction (PCR), which has proved to be a more sensitive method than blood culture,^20^, ^21^ by using blood after colorectal ESD to evaluate whether bacteremia is associated with the origin of fever and PECS.

## METHODS

### Study design and patients

This retrospective analysis of the database on colorectal ESD was conducted at Okayama University Hospital in Japan. The records of 512 consecutive patients, who underwent colorectal ESD between January 2015 and June 2020 were reviewed. The inclusion criteria were as follows: patients >20 years old, having lesions indicated for ESD based on endoscopic findings (lesions with a depth of invasion limited to the mucosa or submucosa, lesions that were difficult to resect en bloc by EMR, recurrent lesions, and residual lesions with non‐lifting sign), the procedure by ESD, or hybrid ESD. The exclusion criterion was cessation of the procedure. Finally, a total of 510 patients were enrolled and analyzed.

The local ethics committee approved the study. This study was conducted according to the Standards for the Reporting of Diagnostic Accuracy Studies initiative and performed in accordance with the Declaration of Helsinki. Informed consent was obtained in the form of an opt‐out on the website.

In addition, we performed broad‐range PCR with 16S rRNA gene analysis when this retrospective study was analyzed. We used 100 serum samples, which could be stored and available just before and the morning after ESD (24 and 76 samples, respectively).

### ESD procedure

The ESD procedure for colorectal neoplasia was performed using a 1.5‐mm DualKnife J (KD‐655Q; Olympus Optical Co., Tokyo, Japan) and a Mucosectome 2 (Pentax‐Hoya Co., Tokyo, Japan). Glycerol (10% glycerol and 5% fructose; Chugai Pharmaceutical Co., Tokyo, Japan), MucoUp (0.4% sodium hyaluronate; Boston Scientific Co., Tokyo, Japan), and a small amount of epinephrine and indigo carmine were injected into the submucosal layer to lift the mucosa. High‐frequency generators (VIO 300D; ERBE Elektromedizin GmbH, Tübingen, Germany) were used. After the tumor removal, clip closure for the mucosal defect was performed according to the endoscopist's decision. Complete closure was defined as no substantial submucosal areas in the closure line. All patients were administered an intravenous dose of 1 g of cefmetazole sodium twice on the day of the ESD and twice on the day after ESD.

### Data analysis

We collected the data as follows: patient age, sex, body mass index, the American Society of Anesthesiologist Physical Status (PS‐ASA) classification system, Charlson comorbidity index (CCI), prognostic nutritional index (PNI), steroids, immunosuppressants (including azathioprine, AZA; 6‐mercaptopurine, 6‐MP; methotrexate, MTX; tacrolimus, TAC; ciclosporin, CyA; mycophenolate mofetil, MMF), antithrombotic agents, tumor morphology (polypoid or granular type laterally spreading tumor, non‐polypoid or non‐granular type laterally spreading tumor), tumor location (cecum to ascending colon or other areas in the colon and rectum), resected specimen size, procedure time of ESD, injury to the muscle layer or perforation during the procedure and delayed perforation, complete closure of mucosal defects, resected multiple lesions, and the clinical course after ESD: abdominal pain, fever, delayed bleeding, delayed perforation, PECS, emergency surgery, and period of hospitalization (days).

Post‐ESD fever was defined as a body temperature ≥37.6°C after ESD until discharge. PECS was defined as localized abdominal tenderness and fever (≥37.6°C) or an inflammatory response (leukocytosis [≥10,000 cells/μl] or a high C‐reactive protein level [≥0.5 mg/dl]) without definite evidence of perforation that occurred ≥6 h after colorectal ESD. Delayed bleeding was defined as apparent bleeding that required emergency endoscopic hemostasis or transfusion or a decrease of >2 g/dl in the hemoglobin level following ESD. Tumor characteristics and treatment details were extracted from the largest lesion when we performed ESD for multiple lesions.

### DNA extraction and broad‐range 16S rRNA PCR

DNA extraction from 1 ml preserved serum was performed using the QIAamp Circulating Nucleic Acid Kit according to the manufacturer's suggestions (Qiagen, Hilden, Germany). A broad‐range PCR assay was performed to amplify the 16S rRNA gene using published primers (530F_‐GCCAGCMGCNGCGGTA‐3_and 1061R_‐CRRCACGAGCTGACGAC‐3). The PCR was performed in 10 μl reactions containing 30 ng of genomic DNA (1 μl), 5 μl of Gflex PCR Buffer, 0.2 μl of Tks Gflex DNA Polymerase (Takara Bio Inc., Otsu, Japan), 0.5 μM forward and reverse primers, and sterile DNase‐and RNase‐free water. The PCR reaction conditions in a thermal cycler were as follows: initial denaturation for 1 min at 94°C and 35 cycles consisting of 10 s at 98°C, 15 s at 50°C, and 15 s at 68°C. The amplified PCR products were electrophoresed on 1% agarose gel and visualized under ImageQuant LAS‐4000 (GE Healthcare, Chalfont St. Giles, UK).

### Measured outcome

The primary endpoint was the detection of the risk factors for post‐ESD fever. The secondary endpoints were (1) to detect the risk factor of PECS, (2) to clarify the postoperative course of patients who showed post‐ESD fever and PECS, and (3) to evaluate the origin of fever by broad‐range 16S rRNA PCR.

### Statistical analysis

The JMP version 14.0 software package (SAS Institute, Cary, NC, USA) was used for all statistical analyses. Continuous variables were expressed as median or mean, standard deviation, and range and assessed by the student's *t*‐test or nonparametric tests. The Pearson's chi‐squared test or Fisher's exact test was performed to compare categorical variables. The odds ratios (ORs) for the prediction of post‐ESD fever and PECS were calculated using logistic regression.

Perforation during the procedure and delayed perforation were excluded when analyzing the risk factors of PECS. The differences were considered significant at a *p*‐value of less than 0.05. We performed multivariate analysis for each factor which showed *p* < 0.05 in univariate analysis.

## RESULTS

### Patients and lesion characteristics

A total of 510 patients with 546 lesions underwent complete colorectal ESD. Two patients whose treatment was discontinued during ESD because of severe fibrosis and severe bleeding with perforation were excluded from this study. The patient and lesion characteristics are shown in Table 1. The number of patients with PS‐ASA ≥3 was 72 (14.1%). A total of 57 patients (11.2%) had CCI ≥2. The number of patients using steroids and immunosuppressants was 23 (4.5%) and 12 (2.3%), respectively. A total of 23 patients (4.5%) used PSL ≥5 mg or immunosuppressants. A total of 203 tumors (39.8%) were located on the right side. The number of patients with multiple lesions was 37 (7.3%).

**TABLE 1:** Patient and tumor characteristics

**Patient characteristics**	***n* = 510**
Age, median (range)	70 (30–90)
Sex, male/female, *n* (%)	272 (53.3)/238 (46.7)
BMI, median (range)	22.2 (14.8–35.2)
PS‐ASA 1/2/3/4, *n* (%)	125 (24.5)/313 (61.4)/72 (14.1)/0 (0)
Charlson comorbidity index 0/1/2‐, *n* (%)	266 (52.2)/187 (36.7)/57 (11.2)
PNI, median (range)	51 (31.5–65.5)
Immunosuppressant agents*, *n* (%)	12 (2.3)
(AZA/ 6‐MP/ MTX/ TAC/ CyA/ MMF)	(2/ 1/ 4/ 5/ 1/ 1)
Steroids; PSL <5mg/ 5mg ≤PSL <10mg/ PSL ≥10mg, *n* (%)	23 (4.5); 10 (2.0)/ 9 (1.8)/ 4 (0.8)
Antithrombotic agents, *n* (%)	72 (14.2)

*Tumor characteristics*	*n = 510* **
Location, right side***, *n* (%)	203 (39.8)
Size, median (range)	30 (6–140)
Morphology, polypoid or LST‐G/non‐polypoid or LST‐NG, *n* (%)	169 (33)/341 (67)
Histologic type, cancer/others, *n* (%)	138 (27)/372(73)
Submucosal invasion, *n* (%)	71 (13.9)

Abbreviations: AZA, azathioprine; BMI, body mass index; CyA, ciclosporin; 6‐MP, 6‐mercaptopurine; LST‐G, granular type laterally spreading tumor; LST‐NG, non‐granular type laterally spreading tumor; MMF, mycophenolate mofetil; MTX, methotrexate; PNI, prognostic nutritional index; PS‐ASA, American Society of Anesthesiologists physical status; PSL, prednisolone; TAC, tacrolimus.

* the number of patients taking one or multiple immunosuppressants.

** tumor characteristic of the largest lesions if treated multiple lesions.

*** right side: cecum and ascending colon.

### Treatment details and clinical course

The treatment details are shown in Table 2. The median specimen size and treatment time were 30 mm and 52 min, respectively. A total of 30 (5.9%) patients had muscle layer injuries. Perforation occurred in 19 patients (3.7%). The complete closure of the mucosal defect was performed in 311 patients (61%). Delayed perforation occurred in five patients (0.9%). Seventy‐five patients (14.7%) experienced abdominal pain and 63 patients (12.4%) experienced fever after ESD. PECS occurred in 43 patients (8.4%). Three cases (two cases, delayed perforation; one case, perforation during the procedure) required emergency surgery after ESD because of peritonitis that could not be improved by observation treatment. All surgeries were performed in 2015, and there were no cases after 2015.

**TABLE 2:** Treatment details and clinical course

**Treatment details**	***n* = 510** *
ESD/hybrid ESD, *n* (%)	457 (89.6)/53 (10.4)
En bloc, *n* (%)	485 (95.1)
Treatment time, median days (range)	52 (5–630)
Muscle injury, *n* (%)	30 (5.9)
Perforation, *n* (%)	19 (3.7)
Complete closure, *n* (%)	311 (61)

*Clinical course*	*n = 510*
WBC, median (range)	6690 (1780–18010)
CRP, median (range)	0.46 (0.01–16)
Delayed bleeding, *n* (%)	9 (1.7)
Delayed perforation, *n* (%)	5 (0.9)
Abdominal pain, *n* (%)	75 (14.7)
Fever, *n* (%)	63 (12.4)
PECS, *n* (%)	43 (8.4)
Emergency surgery, *n* (%)	3 (0.6)
Hospitalization periods, mean days (SD)	4.2 (1.2)

Abbreviations: CRP, C‐reactive protein; ESD, endoscopic submucosal dissection, PECS, post‐ESD coagulation syndrome; SD, standard deviation; WBC, white blood cell.

* treatment details of the largest lesions if treated multiple lesions.

### Risk factor for post‐ESD fever and PECS

We performed logistic regression analysis to determine the risk factors for post‐ESD fever and PECS. In univariate analysis, PS‐ASA ≥3, PNI ≤40, the use of immunosuppressants or PSL ≥5 mg, specimens’ size ≥40 mm, treatment time ≥60 min, injury to muscle layer/perforation, and complete closure for mucosal defect were significantly associated with post‐ESD fever (Table 3). In multivariate analysis, PS‐ASA ≥3 (OR, 3.61; 95% confidence interval [CI], 1.86–6.99), the use of immunosuppressants or PSL ≥5 mg (OR, 2.98; 95% CI, 1.05–8.45), and injury to muscle layer/perforation (OR, 3.11; 95% CI, 1.48–6.51) were significantly associated with post‐ESD fever.

**TABLE 3:** Risk factors for fever after endoscopic submucosal dissection

			**Univariate analysis**			**Multivariate analysis**		
**Variable**	**Category (*n*)**	**Fever, *n* (%)**	**Odds**	**CI (95%)**	***p***	**Odds**	**CI (95%)**	***p***
Age	≥70 years (271)	37 (13.7)	1.30	0.76–2.21	0.342			
	<70 years (239)	26 (10.9)	1					
Sex	male (272)	30 (11.0)	0.77	0.45–1.31	0.331			
	female (238)	33 (13.9)	1					
BMI	≥30 (14)	1 (7.1)	0.54	0.07–4.19	1			
	<30 (496)	62 (12.5)	1					
PS‐ASA	≥3 (72)	21 (29.2)	3.88	2.13–7.07	<.0001	3.61	1.86–6.99	0.0001
	1,2 (438)	42 (9.6)	1					
CCI	≥2 (135)	23 (17)	1.72	0.10–2.99	0.0537			
	0,1 (375)	40 (10.7)	1					
PNI	≤40 (25)	7 (28)	2.90	1.16–7.24	0.0178			
	>40 (473)	56 (11.8)	1					
Immunosuppressants or PSL ≥ 5 mg	yes (23)	7 (30.4)	3.37	1.33–8.54	0.007	2.98	1.05–8.45	0.0397
	no (487)	56 (11.5)	1					
Tumor location	right side* (203)	21 (10.3)	0.73	0.42–1.27	0.262			
	left side, rectum (307)	42 (13.7)	1					
Tumor size	≥40 (108)	22 (20.4)	2.47	1.39–4.40	0.0017	1.78	0.89–3.56	0.102
	<40 (394)	37 (9.4)	1					
Submucosal invasion	yes (71)	10 (14.1)	1.19	0.58–2.47	0.633			
	no (439)	53 (12.1)	1					
Treatment time	≥60 (215)	39 (18.1)	2.61	1.51–4.52	0.0004	1.65	0.82–3.30	0.1605
	<60 (294)	23 (7.8)	1					
Injury to muscle layer/perforation	yes (54)	15 (27.8)	3.27	1.68–6.37	0.0003	3.11	1.48–6.51	0.0027
	no (456)	48 (10.5)	1					
Multiple lesions	yes (37)	7 (18.9)	1.74	0.73–4.14	0.208			
	no (473)	56 (11.8)	1					
Complete closure	yes (311)	29 (9.3)	0.50	0.29–0.85	0.0094	0.78	0.42–1.44	0.422
	no (199)	34 (17.1)	1					

Abbreviations: BMI, body mass index; CCI, Charlson comorbidity index; CI, confidence interval; PNI, prognostic nutritional index; PS‐ASA, American Society of Anesthesiologists Physical Status; PSL, prednisolone.

* right side: cecum and ascending colon.

Table 4 shows the risk factors for PECS. A total of 27 cases were excluded in this analysis (perforation during the procedure, 19 cases; delayed perforation, five cases; lack of laboratory data, three cases). In univariate analysis, the use of immunosuppressants or PSL ≥5 mg, tumor located on the right side, specimen size ≥40 mm, treatment time ≥60 min, injury to the muscle layer, and multiple lesions were significantly associated with PECS. In multivariate analysis, the use of immunosuppressants or PSL ≥5 mg (OR, 3.60; 95% CI, 1.14–11.40), tumors located on the right side (OR, 3.35; 95% CI, 1.65–6.80), treatment time ≥60 min (OR, 4.14; 95% CI, 1.78–9.60), injury to the muscle layer (OR, 3.47; 95% CI, 1.28–9.35), and multiple lesions (OR, 3.61; 95% CI, 1.30–10.04) were significantly associated with PECS.

**TABLE 4:** Risk factors for transient fever and electrocoagulation syndrome after endoscopic submucosal dissection

			**Univariate analysis**			**Multivariate analysis**		
**Variable**	**Category (*n*)**	**PECS, *n* (%)**	**Odds**	**CI (95%)**	***p***	**Odds**	**CI (95%)**	***p***
Age	≥70 years (254)	20(7.9)	0.77	0.41–1.43	0.403			
	<70 years (229)	23(10.4)	1					
Sex	male (256)	19 (7.4)	0.68	0.36–1.27	0.225			
	female (227)	24 (10.6)	1					
BMI	≥30 (13)	2 (15.4)	0.54	0.07–4.19	1			
	<30 (470)	41 (8.7)	1					
PS‐ASA	≥3 (66)	7 (10.6)	1.26	0.53–2.95	0.601			
	1,2 (417)	36 (8.6)	1					
CCI	≥2 (130)	10 (7.7)	0.81	0.39–1.69	0.571			
	0,1 (353)	33 (9.4)	1					
PNI	≤40 (24)	2 (8.3)	0.93	0.21–4.98	1			
	>40 (449)	40 (8.91)	1					
Immunosuppressant or PSL≥5mg	yes (23)	5 (21.7)	3.09	1.09–8.77	0.027	3.60	1.14–11.40	0.029
	no (460)	38 (8.3)	1					
Tumor location	right side* (194)	28 (14.4)	3.08	1.60–5.93	0.0005	3.35	1.65–6.80	0.0008
	left side, rectum (289)	15 (5.2)	1					
Tumor size	≥40 (105)	19 (18.1)	3.35	1.75–6.43	0.0001	1.73	0.81–3.70	0.154
	<40 (372)	23 (6.2)	1					
Submucosal invasion	yes (69)	7 (10.1)	1.19	0.51–2.78	0.695			
	no (414)	36 (8.7)	1					
Treatment time	≥60 (203)	33 (16.3)	5.24	2.52–10.91	<.0001	4.14	1.78–9.60	0.0009
	<60 (280)	10 (3.6)	1					
Injury to muscle layer	yes (30)	8 (26.7)	4.34	1.80–10.46	0.0004	3.47	1.28–9.35	0.014
	no (453)	35 (7.7)	1					
Multiple lesions	yes (33)	7 (21.2)	3.10	1.26–7.63	0.01	3.61	1.30–10.04	0.014
	no (450)	36 (8)	1					
Complete closure	yes (295)	23 (7.8)	0.71	0.38–1.33	0.285			
	no (188)	20 (10.6)	1					

Abbreviations: BMI, body mass index; CI, confidence interval; CCI, Charlson comorbidity index; PECS, post‐ESD coagulation syndrome; PNI, prognostic nutritional index; PS‐ASA, American Society of Anesthesiologists Physical Status; PSL, prednisolone.

* right side: cecum and ascending colon.

### Type of immunosuppressants and the dose of steroid with post‐ESD fever and PECS

Regarding the type of immunosuppressants, three cases of TAC, 1 case of CyA, one case of MTX, and one case of MMF were used in patients with post‐ESD fever, respectively. PECS occurred in two cases of TAC and one case of CyA. Regarding the dose of PSL, three cases of PSL <5 mg, three cases of 5 mg ≤PSL <10 mg, and 0 cases of PSL ≥10 mg were used in patients with post‐ESD fever, respectively. PECS occurred in two cases of PSL <5 mg, three cases of 5mg ≤PSL <10 mg, and 0 cases of PSL ≥10 mg.

### The clinical course of the use of immunosuppressants or PSL ≥5 mg for post‐ESD fever and PECS

In the 63 patients with post‐ESD fever, the duration of improvement was 1—7 days. Among the fever cases, all seven patients taking immunosuppressants or PSL ≥5 mg recovered within 1 day, while 20 out of 56 patients (35.7%) who were not taking immunosuppressants or PSL ≥5 mg improved over 2 days. On the other hand, in the 43 patients with PECS, PECS improved within 1–8 days in the patients not taking immunosuppressants or PSL ≥5 mg but within 1–6 days in patients taking immunosuppressants or PSL ≥5 mg.

Both post‐ESD fever and PECS improved conservatively in patients taking immunosuppressants or PSL ≥5 mg, and no serious complication was observed.

### Broad‐range 16S rRNA PCR

All 24 samples collected before the ESD showed PCR‐negative results. Of the 76 samples collected after ESD, 36 samples (47.3%) were PCR‐positive. Twenty‐one samples (50%) were PCR‐positive among 42 samples with post‐ESD fever, while 15 samples (44.1%) were also PCR‐positive among 34 samples without post‐ESD fever. Seven samples (63.6%) were PCR‐positive among 11 samples with PECS, while 29 samples (44.6%) were also PCR‐positive among 65 samples without PECS. Both post‐ESD fever and PECS were not associated with the result of PCR (post‐ESD fever, *p* = 0.61; PECS, *p* = 0.3).

## DISCUSSION

In this study, we revealed the risk factors for post‐ESD fever and PECS. PS‐ASA ≥3, the use of immunosuppressants or PSL ≥5 mg, and muscle layer injury/perforation were the independent risk factors for post‐ESD fever. On the other hand, the risk factors for PECS were the use of immunosuppressants or PSL ≥5 mg, tumor located on the right side, treatment time ≥60 min, muscle layer injury, and multiple lesions. Although the use of immunosuppressants or PSL ≥5 mg were the risk factors for both complications, these drugs did not lead to severe complications after colorectal ESD. Our results indicated that the ESD for patients taking these drugs was permittable. In addition, our new findings by broad‐range 16S rRNA PCR suggested that bacteremia might occur in the blood after colorectal ESD, but did not associate with the occurrence of post‐ESD fever and PECS.

Post‐ESD fever is one of the most frequent complications after colorectal ESD (4.6%–41%).^12^, ^22^, ^23^ However, few studies have focused mainly on post‐ESD fever. Therefore, the mechanism, risk factors, and clinical course of post‐ESD fever are still unknown. In our study, the rate of post‐ESD fever was 12.4%. Our results showed that negative immune response factors such as PS‐ASA and immunosuppressive drugs might cause post‐ESD fever. Additionally, it is also easy to interpret that muscle damage is a risk factor, as physical damage can cause fever as well. We speculated that both transient enterobacterial infection through mucosal defects and heat coagulation to the submucosal and muscle layers might be the main cause of post‐ESD fever and revealed that bacteremia after ESD was found at a certain frequency by using broad‐range 16S rRNA PCR. However, a causal relationship between fever and bacteremia could not be demonstrated. These results may mean that heat coagulation during the procedure on negative immune conditions is more related to post‐ESD fever than bacterial inflammatory factors from mucosal defects. Our findings that complete closure of mucosal defect did not prevent post‐ESD fever also supported this possibility. However, given the result that negative immune factors were the risk factor of post‐ESD fever, further study is needed on the effect of non‐bacterial inflammatory factors including viruses from mucosal defects.

Bacteremia after an endoscopic procedure has been reported by blood culture, and the endoscopic injection sclerosis of esophageal varices is considered a high‐risk procedure for bacteremia, with a mean of 14.6%.^27^ Min et al. reported that 1 of 40 patients showed a positive blood culture result after EMR.^28^ However, there have been no reports of bacteremia being detected after colorectal ESD. We performed broad‐range 16S rRNA PCR, as a more sensitive method than blood culture, because the blood culture positivity is low; usually 10%–30%.^29^, ^30^ Our results proved that bacteremia occurred in nearly half of the cases after ESD treatment. However, there is no significant relationship between fever and bacteremia. Our results may imply that the highly sensitive method of 16S rRNA PCR detects the extremely low number of bacteria which does not affect post‐ESD complications.

PECS, which is defined as localized abdominal pain or tenderness without signs of perforation on diagnostic imaging, also sometimes occurs after colorectal ESD (8.6%–9.5%),^12^, ^13^, ^24^ and the rate of PECS in our study was 8.4%. This syndrome resembles previously reported “coagulation syndrome (CS),” including post‐polypectomy CS, post‐polypectomy electrocoagulation syndrome, and trans‐mural burn syndrome.^25^ Although the definitions of these syndromes in each study varied, previous reports investigated that the risk factors of these syndromes were the right side of the colon, the size of the tumor, the duration of the procedure, no use of prophylactic antibiotics, and no complete closure of mucosal defects.^12^, ^13^, ^14^, ^22^, ^25^, ^26^ Our study showed consistent results with previous reports, such as tumor location and procedure time. In addition, we revealed a new risk factor for PECS with the use of immunosuppressants and steroids.

Post‐ESD fever with taking immunosuppressants or PSL ≥5 mg is temporary and improved soon. No serious complications of colorectal ESD were observed in patients taking these drugs who showed post‐ESD fever and PECS in this study was also a significant finding. In other words, these drugs have not caused any serious side effects that would require giving up ESD and transferring surgery from the start. Based on these findings, we believe that endoscopists might be permitted to perform ESD for patients taking these drugs if we understand the effects of these drugs on colorectal ESD. Although there has been no study to discuss the association between immunosuppressants and complications of colorectal ESD, this is because colorectal ESD for patients taking these drugs might often be avoided in general hospitals and most clinical trials also exclude these patients.

To interpret immunosuppressants and steroids as the risk factor of post‐ESD fever and PECS, we evaluated the relationship depending on the type of immunosuppressants and the dose of steroid. Calcineurin inhibitors (CNI), such as TAC and CyA, reduce the release of cytokine interleukin‐2 from the T cells and T‐cell proliferation. Antimetabolites, such as AZA, 6‐MP, MTX, and MMF, inhibit the synthesis of purine or pyrimidine nucleotide or by competing with them in DNA or RNA synthesis. Post‐ESD fever and PECS occurred in four (66.7%) and three patients (50%) among the six patients taking CNI, while in two (25%) and no patients (0%) among the eight patients taking antimetabolites. Although we did not show a significant difference between the two drugs due to the small number of patients, CNI which has a strong immune‐suppress effect showed a high tendency. On the other hand, all four patients taking PSL ≥10 mg did not show post‐ESD fever and PECS, which might imply post‐ESD fever and PECS did not occur in dose dependency. Additional study is needed to confirm the efficacy and prove the mechanism of these drugs in colorectal ESD.

The limitations of this study need to be mentioned. First, this study was a retrospective review of patients treated at a single institution. The number of patients taking immunosuppressants and PSL were comparatively small; however, these drugs were an independent risk factor for both post‐ESD fever and PECS using multivariate analysis. To validate our findings, a further prospective, cohort study for the effectiveness of immunosuppressants and PSL in colorectal ESD will be needed in the future. Second, the mechanism that immunosuppressants and PSL occur post‐ESD fever and PECS is unclear. Immune deficiency by these drugs might prevent the resistance to inflammation factors from heat coagulation and mucosal defect. Basic research is also needed. Third, we used antimicrobial prophylaxis in all patients. Therefore, we could not analyze the effect of antimicrobial prophylaxis for post‐ESD fever and PECS. In addition, antimicrobial prophylaxis might affect the results of broad‐range 16S rRNA PCR. Fourth, the breadth of broad‐range 16S rDNA PCR is vulnerable to contamination due to the high sensitivity for bacteremia. However, our results for all 24 samples collected before ESD were PCR‐negative.

In conclusion, we revealed the risk factors for post‐ESD fever and PECS; additionally, the use of immunosuppressants or PSL ≥5 mg were the risk factors for both complications. However, colorectal ESD might be permittable with no severe complications for patients taking these drugs, although broad‐range 16S rRNA PCR suggested that bacteremia might occur after colorectal ESD with or without post‐ESD fever and PECS.

## CONFLICT OF INTEREST

The authors declare that they have no conflict of interest.

## FUNDING INFORMATION

This study was supported by JSPS KAKENHI (19k17433).
